# Abnormalities of Inter- and Intra-Hemispheric Functional Connectivity in Autism Spectrum Disorders: A Study Using the Autism Brain Imaging Data Exchange Database

**DOI:** 10.3389/fnins.2016.00191

**Published:** 2016-05-03

**Authors:** Jung Min Lee, Sunghyun Kyeong, Eunjoo Kim, Keun-Ah Cheon

**Affiliations:** ^1^Division of Computational Mathematics, National Institute for Mathematical SciencesDaejeon, South Korea; ^2^Severance Biomedical Science Institute, Yonsei University College of MedicineSeoul, South Korea; ^3^Division of Child and Adolescent Psychiatry, Department of Psychiatry and Institute of Behavioral Science in Medicine, Yonsei University College of MedicineSeoul, South Korea

**Keywords:** Autism Spectrum Disorder (ASD), inter-hemisphere, intra-hemisphere, functional connectivity, resting state fMRI

## Abstract

Recently, the Autism Brain Imaging Data Exchange (ABIDE) project revealed decreased functional connectivity in individuals with Autism Spectrum Disorders (ASD) relative to the typically developing controls (TDCs). However, it is still questionable whether the source of functional under-connectivity in subjects with ASD is equally contributed by the ipsilateral and contralateral parts of the brain. In this study, we decomposed the inter- and intra-hemispheric regions and compared the functional connectivity density (FCD) between 458 subjects with ASD and 517 TDCs from the ABIDE database. We quantified the inter- and intra-hemispheric FCDs in the brain by counting the number of functional connectivity with all voxels in the opposite and same hemispheric brain regions, respectively. Relative to TDCs, both inter- and intra-hemispheric FCDs in the posterior cingulate cortex, lingual/parahippocampal gyrus, and postcentral gyrus were significantly decreased in subjects with ASD. Moreover, in the ASD group, the restricted and repetitive behavior subscore of the Autism Diagnostic Observation Schedule (ADOS-RRB) score showed significant negative correlations with the average inter-hemispheric FCD and contralateral FCD in the lingual/parahippocampal gyrus cluster. Also, the ADOS-RRB score showed significant negative correlations with the average contralateral FCD in the default mode network regions such as the posterior cingulate cortex and precuneus. Taken together, our findings imply that a deficit of non-social functioning processing in ASD such as restricted and repetitive behaviors and sensory hypersensitivity could be determined via both inter- and intra-hemispheric functional disconnections.

## Introduction

Autism Spectrum Disorder (ASD) is characterized by impairments in social interaction and communication, and restrictive and repetitive behaviors (American Psychiatric Association, 2013). ASD is caused by genetic and neurobiological factors (Frith and Happe, 1993; Bogdashina, 2006; Hughes, 2009; Amaral, 2011). Among the neurobiological mechanisms, the prevailing theory is that ASD is caused by abnormalities in the neuronal system and social brain network (Bogdashina, 2006; Minshew and Keller, 2010; Nebel et al., 2014). In functional neuroimaging studies, researchers have investigated the functional connectivity in subjects with ASD (Just et al., 2007), and the under-connectivity theory of ASD has been supported by many previous studies (Muller et al., 2011; Rudie et al., 2012; Vissers et al., 2012; Di Martino et al., 2014). A neuronal network study using electroencephalography showed long-range under-connectivity and short-range over-connectivity in patients with ASD (Barttfeld et al., 2011). The white matter integrity of the brain network has been examined with diffusion tensor imaging analysis. Furthermore, a decreased size of the corpus callosum was observed in the ASD group in a structural magnetic resonance imaging (MRI) study (Keary et al., 2009). Because the corpus callosum is the biggest part of the transcallosal connectivity in the human brain, a decreased corpus callosum size has been argued as an indication of long-range under-connectivity. Moreover, decreased volumes and abnormal integrity of the corpus callosum have been observed in diffusion tensor imaging studies (Alexander et al., 2007; Keller et al., 2007; Cheon et al., 2011).

Many researchers utilized the resting state functional MRI and found atypical brain activities in ASD (Cherkassky et al., 2006; Di Martino et al., 2014). Due to the restricted computational power, studies in the neuroimaging, and psychiatric research areas predominantly used a seed-based analysis by considering a default network alteration (Monk et al., 2009; Weng et al., 2010). Recently, voxel-wise data-driven functional connectivity density (FCD) mapping method was proposed (Tomasi and Volkow, 2010), in which all voxels in the whole brain region would be examined rather than using a specific seed region of interest for a connectivity analysis. The FCD method has been applied successfully to analyze the sex differences in functional connectivity in both healthy control subjects (Tomasi and Volkow, 2012b) and subjects with attention deficit hyperactivity disorder (ADHD) using large samples (Tomasi and Volkow, 2012a).

Due to the diverse symptoms and complexity of ASD, a large dataset is needed to investigate the characteristic features of ASD. Recently, the Autism Brain Imaging Data Exchange (ABIDE) collected 1112 resting state functional MRI datasets of subjects with ASD and typically developing control (TDC) subjects from 17 international sites. Previously, large datasets from multiple centers have been successfully analyzed to identify features of the functional human brain (Lord et al., 2000; Biswal et al., 2010; Tomasi and Volkow, 2012a). In particular, the FCD method was applied successfully to the ADHD-200 dataset to identify differences in the functional hubs among children with ADHD (Tomasi and Volkow, 2012a). A deeper understanding of the connectivity abnormalities in ASD has been achieved with the worldwide neuroimaging data sharing initiative. Di Martino et al. analyzed brain connectivity in 1000 subjects using global connectivity measures, and revealed over- and under-connectivity in the ASD group (Di Martino et al., 2014).

Several studies have examined the regional inter-hemispheric under-connectivity in ASD by evaluating the correlation between a voxel and its opposite hemispheric counterpart on a symmetric template (Anderson et al., 2011; Di Martino et al., 2014). However, the whole-brain inter- and intra-hemispheric functional under-connectivity studies have been rarely investigated to uncover the deficit of social and non-social functioning in ASD. Because the global FCD was originally proposed to identify the functional hub regions, the ipsilateral FCD and contralateral FCD measure the intra-hemispheric and inter-hemispheric functional hub regions, respectively. A recently published study using the ABIDE datasets examined both intra-hemispheric and inter-hemispheric connectivity in participants with ASD, and revealed both increased and decreased connectivity in the ASD groups in comparison to the control groups, depending on the different types of brain connectivity and distortions in connectivity patterns examined at the individual level (Hahamy et al., 2015). In the current study, we aimed to examine both inter- and inter-hemispheric connectivity in the brains of participants with ASD by decomposing the functional connectivity into ipsilateral and contralateral parts. These approaches would allow us to investigate how connectivity abnormalities in the ASD group would be determined by interactions between the intra- and inter-hemispheric functional connectivity. The results would also confirm that the abnormal regions of the resting state functional connectivity in ASD are related to the default mode network (Raichle and Snyder, 2007; Assaf et al., 2010; Weng et al., 2010; Lynch et al., 2013) and brain regions implicated in the non-social functioning processes such as restricted and repetitive behaviors (Di Martino et al., 2009; Supekar et al., 2013; von dem Hagen et al., 2013; Fishman et al., 2014).

## Materials and methods

### Dataset

We used resting state fMRI data of 458 subjects with ASD and 517 TDCs (see Table 1 for additional demographic information) among 1112 datasets from the ABIDE datasets (http://fcon_1000.projects.nitrc.org/indi/abide). To minimize the institution dependent variability, our study included data from research centers that contributed to both the ASD and TDC groups. Therefore, the present study included neuroimaging datasets from California Institute of Technology (CALTECH), Kennedy Krieger Institute (KKI), Ludwig Maximilians University, Munich (MAX_MUM), New York University Langone Medical Center (NYU), Olin Institute of Living at Hartford Hospital (OLIN), Oregon Health & Science University (OHSU), University of Pittsburgh School of Medicine (PITT), San Diego State University (SDSU), Stanford University (STANFORD), Trinity Center for Health Sciences (TRINITY), University of California, Los Angeles (UCLA), University of Leuven (LEUVEN), University of Michigan (UM), University of Utah School of Medicine (USM), and Yale Child Study Center (YALE). All experimental protocols were in compliance with the policies of site-specific institutional review boards. The demographic variables and scanning parameters are summarized in Table 1. The graphical illustration of the demographic variables can be found in elsewhere (Di Martino et al., 2014). We excluded datasets from the social brain Lab BCN NIC UMC Groningen and Netherlands Institute for Neurosciences due to the missing information of a full-scale intelligence quotient (IQ) for many subjects. After preprocessing, we found that a large part of the cerebellum was missing in the imaging dataset of Carnegie Mellon University, and all datasets from Carnegie Mellon University were excluded. To control for potential confounding effects of IQ in our analysis, we decided to exclude five datasets that included some subjects with IQ lower than 70. Finally, we performed voxel-wise group comparisons with 975 imaging datasets.

**Table 1:** **Demographic variables and imaging parameters for the selected resting-state functional MRI datasets from Autism Brain Imaging Data Exchange database**.

**Center**	**No. of scans**	**TR(ms)**	**TDC**			**ASD**			
			**M/F**	**Age**	**FIQ**	**M/F**	**Age**	**FIQ**
PITT	200	1500	23/4	18.9 ± 6.5	110.1 ± 9.2	26/4	18.9 ± 7.1	110.0 ± 14.1
OLIN	210	1500	14/2	16.9 ± 3.6	114.9 ± 16.0	16/2	16.3 ± 3.0	113.0 ± 17.4
OHSU	82	2500	15/0	10.1 ± 1.0	115.7 ± 10.7	10/0	10.9 ± 1.8	109.7 ± 18.4
SDSU	180	2000	16/6	14.2 ± 1.9	108.1 ± 10.3	13/1	14.7 ± 1.7	111.4 ± 17.4
TRINITY	150	2000	25/0	17.1 ± 3.7	110.9 ± 12.0	24/0	17.3 ± 3.5	109.3 ± 14.7
UM	300	2000	56/17	14.6 ± 3.6	108.0 ± 9.7	50/9	13.2 ± 2.5	107.3 ± 16.8
USM	240	2000	43/0	21.4 ± 7.6	115.1 ± 13.6	57/0	22.4 ± 7.5	100.9 ± 15.2
YALE	200	2000	20/8	12.7 ± 2.7	105.0 ± 17.1	18/8	12.7 ± 3.0	97.9 ± 17.8
LEUVEN	250	1667	15/0	23.3 ± 2.8	114.8 ± 12.4	14/0	21.9 ± 4.0	109.4 ± 12.6
KKI	156	2500	23/9	10.1 ± 1.2	113.8 ± 8.9	16/4	10.0 ± 1.5	97.8 ± 16.4
NYU	180	2000	79/26	15.8 ± 6.2	113.2 ± 13.1	68/10	14.5 ± 7.0	108.1 ± 16.5
STANDFORD	180	2000	16/4	10.0 ± 1.6	112.1 ± 15.0	16/3	9.9 ± 1.5	113.3 ± 17.5
UCLA	120	3000	38/6	13.0 ± 1.9	106.3 ± 10.8	46/6	13.1 ± 2.4	100.9 ± 13.2
MAX_MUN	120	3000	29/4	26.2 ± 9.7	111.5 ± 8.7	16/3	22.9 ± 14.1	107.6 ± 13.7
CALTECH	150	2000	15/4	28.9 ± 10.9	114.2 ± 9.4	14/4	27.8 ± 10.2	108.2 ± 12.2
Total			427/90	16.5 ± 7.3	111.2 ± 12.4	404/54	16.2 ± 7.4	106.0 ± 16.3

*ASD, autism spectrum disorder; CALTECH, California Institute of Technology; F, female; FIQ, full scale IQ standard score; KKI, Kennedy Krieger Institute; M, Male; MAX_MUM, Ludwig Maximilians University Munich; NYU, New York University Langone Medical Center; OLIN, Olin Institute of Living at Hartford Hospital; OHSU, Oregon Health and Science University; PITT, University of Pittsburgh School of Medicine; SDSU, San Diego State University; STANFORD, Stanford University; TDC, Typically developed control group; TRINITY, Trinity Center for Health Sciences; UCLA, University of California, Los Angeles; LEUVEN, University of Leuven; UM, University of Michigan; USM, University of Utah School of Medicine; YALE, Yale Child Study Center*.

### Image preprocessing

Datasets were preprocessed with SPM8 (http://www.fil.ion.ucl.ac.uk/spm/software/spm8/). The first step was realignment for head motion correction. Images were realigned to the first image and a mean echo planar image (EPI) was created during this step. Subsequently, structural T1 images were coregistered to their mean EPI data. Registered EPI data of each subject were normalized to Montreal Neurological Institute (MNI) template and spatially smoothed with 8 mm of full-width at half-maximum. Then, in the temporal domain, the linearly increasing trend due to heat absorption was removed at each voxel, and the effects of the head motion, white matter, cerebrospinal fluid, and global signal were regressed out. Lastly, temporal band-pass filtering was applied (0.01–0.08 Hz).

### Functional connectivity density mapping

**Figure 1:**
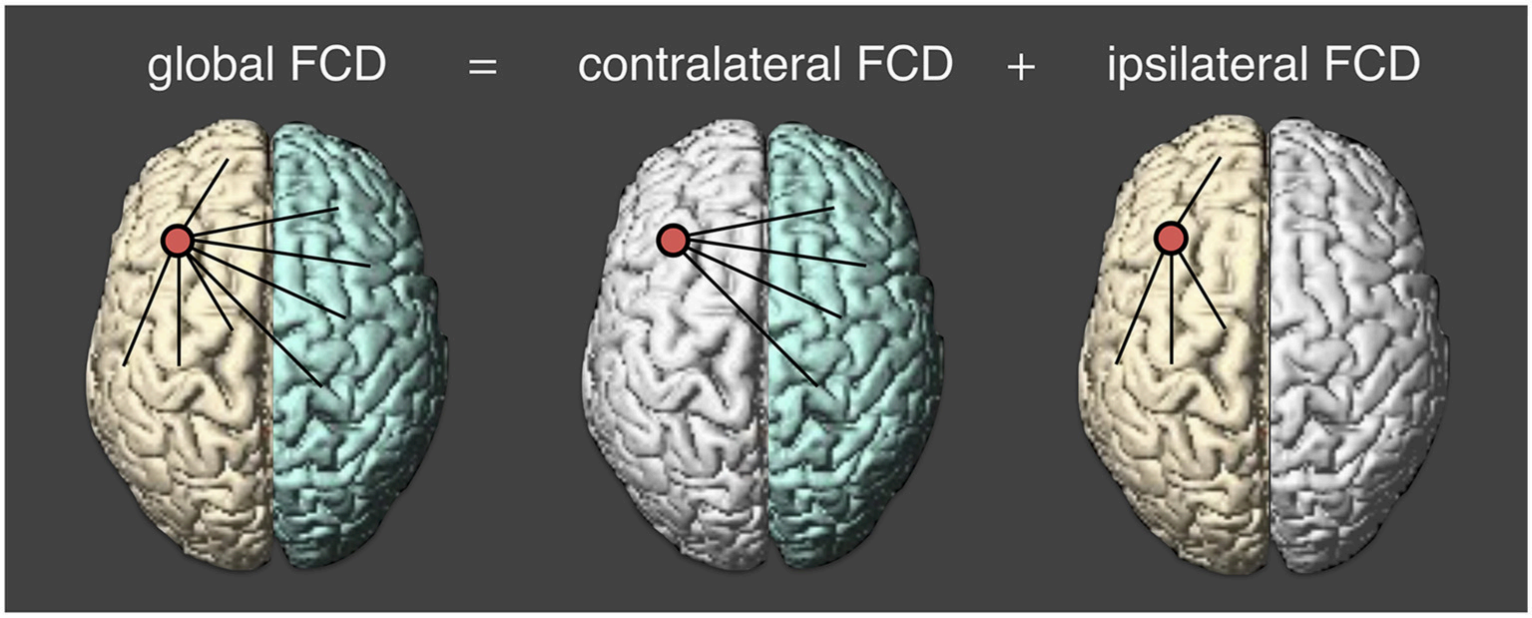
**Calculating three measures of functional connectivity density**. cFCD (contralateral functional connectivity density) is the number of functional connectivity from the opposite hemisphere; iFCD (ipsilateral functional connectivity density) is from the same hemisphere; gFCD (global functional connectivity density) is computed as the sum of cFCD and iFCD.

Preprocessed resting state fMRI data were normalized into the MNI template space with a voxel size of 2 × 2 × 2 mm. At each voxel, we applied a voxel-wise data-driven FCD mapping method to calculate the global FCD, which was introduced by Tomasi and Volkow (2010). In this study, we divided the global functional connectivity density into two parts. One is the contralateral FCD and another is the ipsilateral FCD (Figure 1). The global FCD is the number of functional edges connected with all other voxels. For a given voxel *i*, a voxel *j* is said to be connected to the voxel *i* if the correlation coefficient between *i*-th and *j*-th time series is larger than 0.6 (Tomasi and Volkow, 2010, 2012a,b,c). Then the degree between the two voxels is defined to be 1 (*S*_*ij*_ = 1); and otherwise, the degree is zero (*S*_*ij*_ = 0). Likewise, we could obtain the degree from other voxels and the sum of all degrees at a given voxel *i* is defined as the global FCD at that voxel. At a voxel *i*, the global FCD is calculated as,

$$\begin{array}{l} {\text{global FCD}}_{i}=\overset{N}{\underset{j=1}{\sum }}S_{ij}, \end{array}$$

where *N* is the number of voxels (or nodes) in the gray matter regions.

The contralateral (or inter-hemispheric) FCD is evaluated by counting the number of functional connectivity with all voxels in the opposite hemispheric brain regions as follows:

$$\begin{array}{l} {\text{contralateral FCD}}_{i}=\overset{N}{\underset{j=1}{\sum }}h_{ij}S_{ij}, \end{array}$$

where *h*_*ij*_ is 1 if *j*-th voxels are in the opposite hemispheres and 0 otherwise.

Lastly, the ipsilateral (or intra-hemispheric) FCD was computed by subtracting the number of contra-lateral functional connectivity from the total number of functional connectivity as follows:

$$\begin{array}{l} {\text{ipsilateral FCD}}_{i}={\text{global FCD}}_{i}-{\text{contralateral FCD}}_{i}. \end{array}$$

The graphical illustrations of global, contralateral, and ipsilateral functional connectivity densities are presented in Figure 1. Finally, the normalized contralateral FCD (cFCD), normalized ipsilateral FCD (iFCD), and normalized global FCD (gFCD) were obtained by normalizing the number of functional connections at each voxel with respect to the average value of the global functional connectivity of each subject.

$$\begin{array}{l} {\text{iFCD}}_{i}=\frac{{\text{ipsilateral FCD}}_{i}}{mean\;\left(\text{global FCD}\right)},{\text{cFCD}}_{i}=\frac{{\text{contralateral FCD}}_{i}}{mean\;\left(\text{global FCD}\right)},\\ \text{ and}{\text{ gFCD}}_{i}=\frac{{\text{global FCD}}_{i}}{mean\;\left(\text{global FCD}\right)}, \end{array}$$

where *mean*(global FCD) is the average of the global FCD for all voxels.

The use of the normalized functional connectivity density minimizes the individual variability of the overall connectivity and makes it easier to detect the fractionally increased or decreased connectivity. Consequently, the average of the gFCD becomes one.

### Statistical analysis

The images of the gFCD, cFCD, and iFCD were used for a second-level analysis, comparing the subjects with ASD and TDCs using SPM8, in which a 2-tailed *t*-test design with two covariates of age and sex was used to compute the statistical significance of the FCD differences between the two groups. Statistical significance was based on the false discovery rate corrected *P* < 0.05 together with requiring 20 voxels for the minimum continuous voxel size within a cluster (corrected *P* < 0.05). For brain regions showing significantly decreased FCDs, we computed the partial correlations between the averages (or regional maximum) of the FCD values for each cluster and the scores from symptom severity scales while controlling for effects of age and sex. The Benjamini-Hochberg procedure was applied to control multiple comparison corrections (Benjamini and Hochberg, 1995). The significances of the correlation analysis were set at a threshold of (Benjamini–Hochberg) corrected *P* < 0.05.

## Results

### Demographic variables and clinical measures

We included data from 458 subjects (54 females) with ASD and 517 TDCs (90 females) in the data analysis. The two-sample *t*-test showed that the age distribution of subjects with ASD were not significantly different from that of TDCs, but the full-scale IQ score was significantly lower in the ASD group than that in the TDC group (*P* < 0.05).

### Functional connectivity density

**Figure 2:**
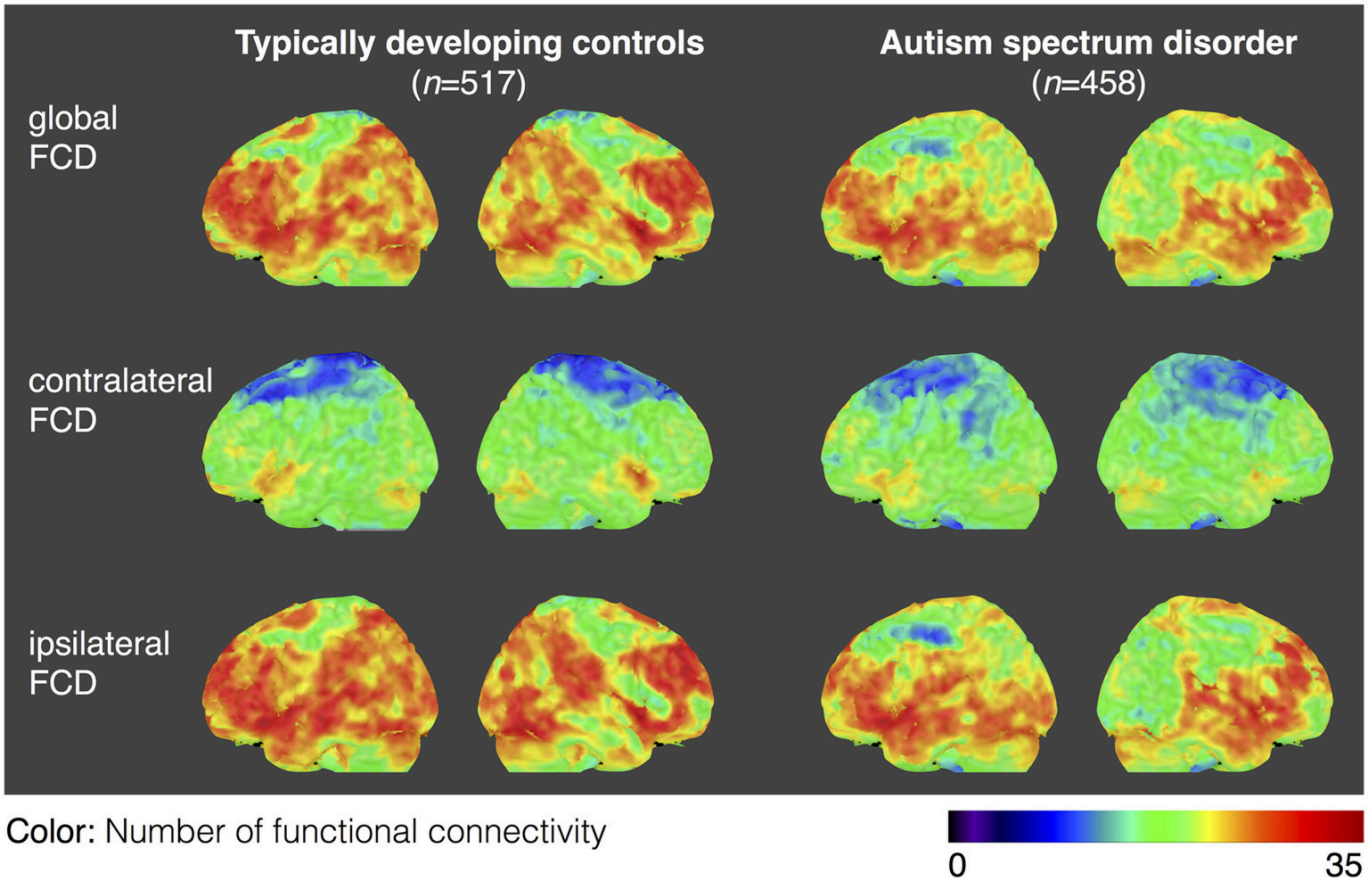
**Average maps of functional connectivity densities for the typically developing controls (TDCs) and autism spectrum disorders (ASDs)**. Distribution of gFCD, cFCD, and iFCD are averaged over 458 patients with ASDs and 517 TDCs. The color bar represents the number of functional connectivities.

**Figure 3:**
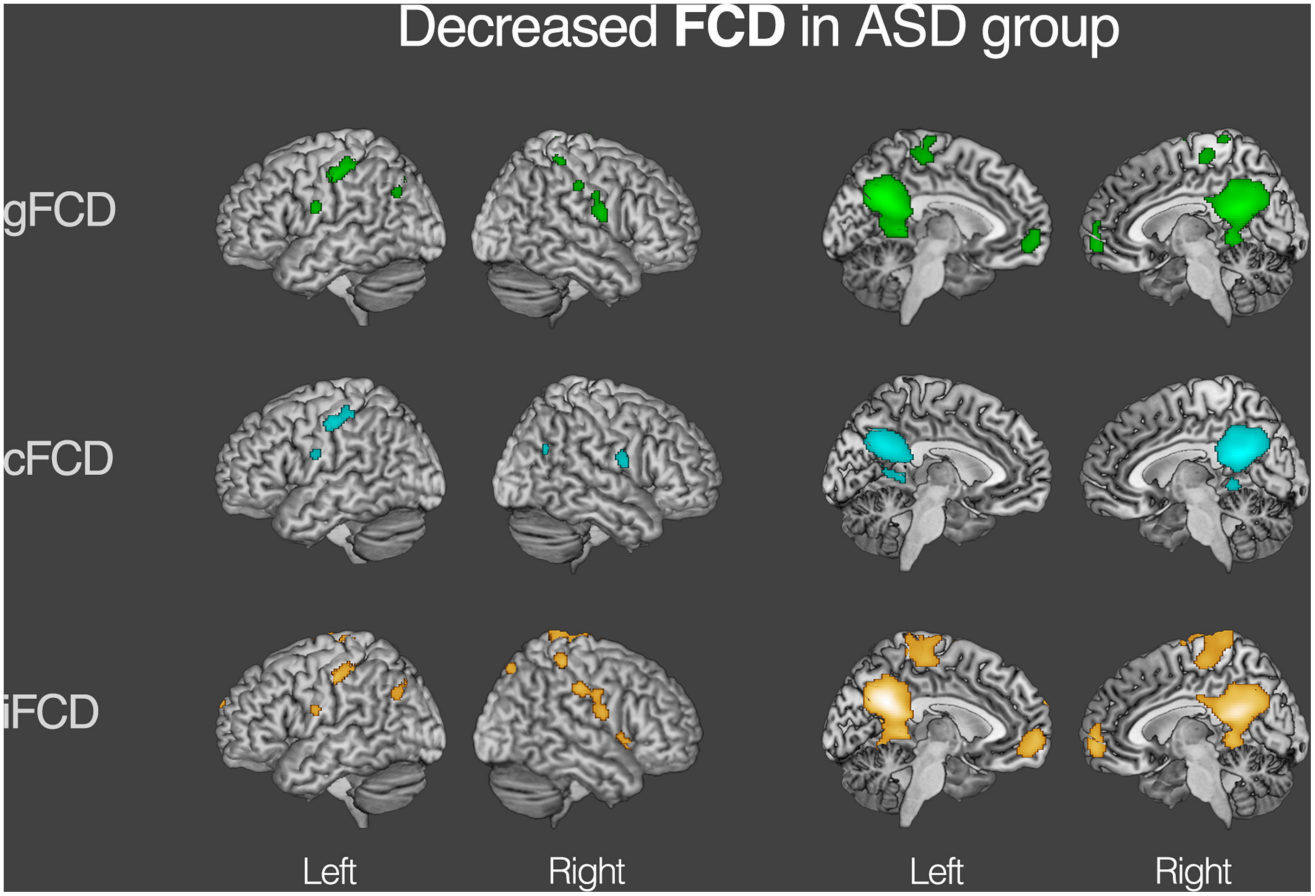
**Decreased functional connectivity density (FCD) in autism spectrum disorder (ASD) group**. Group differences in the normalized global, contralateral, and ipsilateral FCDs were visualized with the statistical significance at corrected *P* < 0.05. Detailed information on each cluster is written in Table 2.

For both the ASD and TDC groups, the average values of gFCD, iFCD, and cFCD are presented (Figure 2), and the distribution of the functional connectivity density were bilateral. For each measure of the normalized gFCDs, iFCDs, and cFCDs, the two-sample *t*-test with covariates of age and sex found significantly decreased functional connectivity in multiple regions in the ASD group, but no significantly increased functional connectivity was detected (Figure 3 and Table 2). For example, in the comparisons of gFCD, the ASD group showed a significantly decreased functional connectivity in the default mode network regions including the medial prefrontal cortex, posterior cingulate cortex, and inferior parietal lobule, and the sensorimotor regions including the bilateral postcentral gyri, paracentral lobule, and parahippocampal gyrus (corrected *P* < 0.05; Table 2 gFCD; Figure 3). The group differences in the iFCD showed similar patterns to that of the gFCD. Furthermore, iFCDs of the right inferior frontal gyrus, left superior frontal gyrus, and precuneus were significantly decreased in the ASD group (corrected *P* < 0.05; Table 2 iFCD, Figure 3). Finally, the comparison of cFCD revealed regional under-connectivity of the ASD group in the posterior cingulate cortex, parahippocampal gyrus, precentral gyrus, and right angular gyrus (corrected *P* < 0.05; Table 2 cFCD, Figure 3).

**Table 2:** **Group differences in the normalized global, contralateral, and ipsilateral FCDs (gFCD, cFCD, and iFCD) between the ASDs and TDCs**.

**Cluster Name**	**BA**	**gFCD**	**cFCD**	**iFCD**
		Cluster Size (MNI)	Cluster Size (MNI)	Cluster Size (MNI)
Lt. Medial prefrontal cortex^*^	10	303 (0, 62, −6)		538 (0, 62, −6)
Lt. Superior frontal gyrus	10			58 (−14, 72, 28)
Rt. Inferior frontal gyrus	22			60 (54, 16, −4)
Lt. Supplementary motor area	6			49 (0, −6, 76)
Lt. Precentral gyrus	4	51 (−64, −4, 22)	29 (−62, −4, 22)	25 (−64, −4, 22)
Lt. Postcentral gyrus	2	648 (−64, −20, 50)	751 (−60, −18, 48)	387 (−64, −20, 48)
Rt. Postcentral gyrus	2	136 (40, −36, 58)	91 (66, −2, 16)	234 (44, −34, 60)
Rt. Lingual/parahippocampal gyrus	30		74 (12, −44, −2)	
Lt. Cerebellum (Culmen)	29	4014 (−6, −56, 28)	113 (−6, −44, 2)	4190 (−6, −56, 28)
Rt. Posterior cingulate cortex^*^	23		2682 (6, −50, 22)	
Rt. Precuneus	7			127 (24, −76, 56)
Lt. Angular gyrus^*^	39	43 (−54, −68, 32)		176 (−46, −78, 46)
Lt. Inferior parietal lobule^*^	39	46 (−48, −76, 46)		
Rt. Angular gyrus	39		29 (50, −62, 26)	
Rt. Supramarginal gyrus	40	24 (62, −22, 42)		224 (62, −22, 40)
Lt. Paracentral lobule	6	333 (0, −24, 60)		1338 (0, −24, 60)
Rt. Paracentral lobule	6	56 (8, −38, 76)		

*The number of voxels and the corresponding center of mass in MNI for each cluster were described accordingly*.

*Cortical clusters that cover wide range of brain areas were highlighted in dark gray*.

Asterisk(

* *) indicates the default mode network regions*.

*BA, Brodmann areas; Lt, center; Rt, right*.

**Figure 4:**
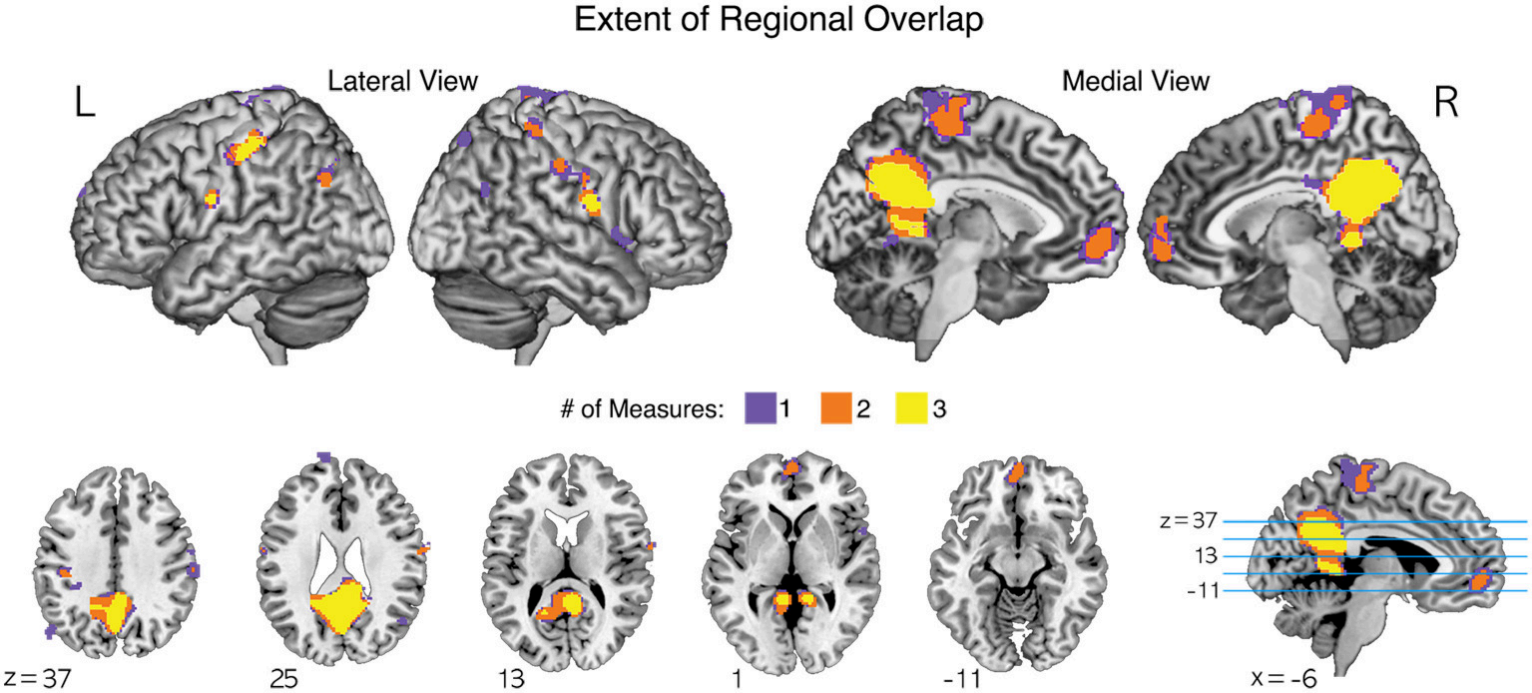
**The extent of regional overlaps for functional underconnectivities in the normalized global, contralateral, and ipsilateral FCDs (gFCD, cFCD, and iFCD)**. Yellow color represents the overlapping areas for functional underconnectivities observed in gFCD, cFCD, and iFCD. Orange color represents the overlapping areas of functional underconnectivities observed in any pairs of FCDs: gFCD and cFCD, gFCD and iFCD, or cFCD and iFCD. Purple color represents functional underconnectivities observed in gFCD, cFCD, or iFCD without overlapping areas.

In particular, common brain regions showing under-connectivity in ASD across different institutions were the default mode network regions, such as the medial prefrontal cortex, posterior cingulate cortex, precuneus, and lingual/parahippocampal gyrus. The extent of regional overlaps in the normalized global, contralateral, and ipsilateral FCDs are presented in Figure 4.

### Correlation between clinical data and FCD

**Figure 5:**
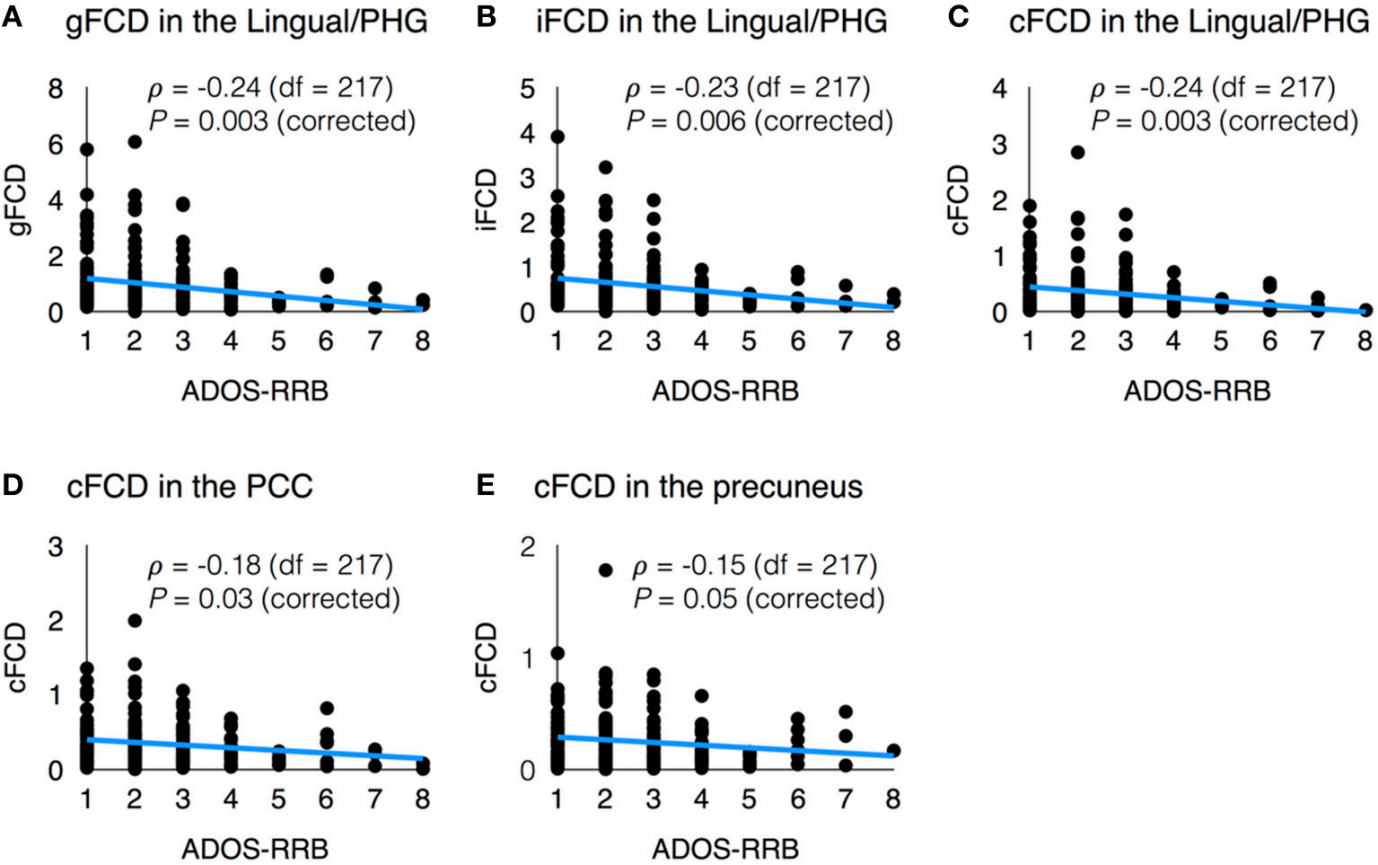
**Scatter plots of functional connectivity density (FCD) vs. clinical variable**. **(A)** gFCD in the Lingual/PHG and ADOS-RRB score; **(B)** iFCD in the Lingual/PHG and ADOS-RRB score; **(C)** cFCD in the Lingual/PHG and ADOS-RRB score; **(D)** cFCD in the PCC and ADOS-RRB score; and **(E)** cFCD in the precuneus and ADOS-RRB score.

In several sites, the datasets contained clinical information such as the Autism Diagnostic Interview-Revised (ADI-R), the restricted and repetitive behavior subscore of the Autism Diagnostic Observation Schedule (ADOS-RRB), and communication subscore of the ADOS (ADOS-COM). The average values of FCDs in each region of interest showed meaningful correlations with the ADI-R scores (Lord et al., 1994) and ADOS scores (Lord et al., 2000). Figure 5 shows the significant correlations between the FCDs and the ADOS-RRB score. In the ASD group, the ADOS-RRB score showed significant negative correlations with the average gFCD (ρ = −0.24, corrected *P* = 0.003, *df* = 217), iFCD (ρ = −0.23, corrected *P* = 0.006, *df* = 217), and cFCD (ρ = −0.24, corrected *P* = 0.003, *df* = 217) in the lingual/parahippocampal gyrus cluster. Also, the ADOS-RRB score showed significant negative correlations with the average cFCD in the PCC (ρ = −0.18, corrected *P* = 0.03, and *df* = 217) and precuneus (ρ = −0.15, corrected *P* = 0.05, and *df* = 217). Moreover, significances of those correlations were preserved if we use a regional maximum value of FCDs instead of a regional mean within each region of interest (corrected *P* < 0.05).

## Discussion

Abnormalities in the neuronal systems of individuals with ASD have been reported in many studies over the last two decades, using small sample ASD groups. We used data from 517 TDCs and 458 patients with ASD from the ABIDE database to examine the abnormalities of functional connectivity in the ASD group relative to TDCs. We used a data-driven voxel-wise method, which examined all voxels in the entire brain. We computed the FCD maps by calculating the connectivity degree of each voxel with all other voxels. The FCD maps were used to compare the functional connectivity between the ASD and TDC groups. A group difference analysis showed regional under-connectivity in the ASD group relative to the TDC group. The abnormal regions of intrinsic functional connectivity in the ASD group were the lingual/parahippocampal gyrus, posterior cingulate cortex, precuneus, postcentral gyrus, paracentral lobule, medial prefrontal cortex, precentral gyrus, angular gyrus, inferior frontal gyrus, superior frontal gyrus, and supplementary motor area, which are related to the default mode network and social functioning processes.

To the best of our knowledge, the present study is the first attempt to decompose the functional connectivity into ipsilateral and contralateral parts to explore abnormalities in the intrinsic neural networks of ASD. Inter-hemispheric connectivity in ASD was examined previously in several studies (Anderson et al., 2011), in which a correlation between each voxel and a corresponding voxel in the opposite hemisphere was calculated. The present study used all voxels in the opposite hemisphere to calculate the inter-hemispheric connectivity. Consequently, inter-hemispheric FCD is somewhat analogous to long distance connectivity. Intra-hemispheric FCD was used to identify the characteristics of the short distance connectivity. Global functional connectivity density was obtained by combining the inter- and intra-hemispheric FCDs.

### Functional under-connectivity vs. over-connectivity

Neuroimaging studies have shown abnormal brain networks in ASD, but the issue of over-connectivity or under-connectivity in those findings remains controversial (Muller et al., 2011). Children with ASD showed both over-and under-connectivity (Lynch et al., 2013). The precuneus was underconnected with other brain regions, as we observed in this study, but the posterior cingulate cortex was overconnected, which was the opposite result from our study. For adolescents with ASD, functional connectivity between the medial prefrontal cortex and precuneus (theory of mind regions) and some regions in the mirror neuron system have been found to be overconnected (Northoff et al., 2006; Fishman et al., 2014). However, when the brains of adults with ASD were examined, the medial prefrontal cortex and precuneus were underconnected. This result may be explained by two reasons. First, including adult subjects may change the overall result from over-connectivity to under-connectivity. However, it is unlikely that the inclusion of adult subjects may change the connectivity pattern of subjects with ASD because the majority of subjects with ASD in our study were children and adolescents. Second, the medial prefrontal cortex and precuneus were overconnected with some regions in the mirror neuron system but underconnected with a majority of other brain regions. In summary, the medial prefrontal cortex and precuneus may appear underconnected in some specific brain regions.

In a prior study where the posterior cingulate cortex had been used as a single seed (Monk et al., 2009), the ASD group showed under-connectivity in the right superior frontal gyrus and over-connectivity in the right temporal pole and right parahippocampal gyrus, compared to the TDC group. In our results using the voxel-wise data-driven method, both the posterior cingulate cortex and parahippocampal gyrus in the ASD group showed reduced functional connectivity compared to TDCs. This result is consistent with the result from prior studies showing a weaker connectivity between the posterior cingulate cortex and precuneus in the ASD group compared to that of the TDC group (Cherkassky et al., 2006).

### Hypofunctional connectivity density

Functional connectivity differences between the ASD and TDC groups were found in the medial prefrontal cortex, posterior cingulate cortex, precuneus, and parahippocampal gyrus. The medial prefrontal cortex, posterior cingulate cortex, and precuneus have been reported to be an important parts of the default mode network, and abnormalities in the default mode network have been reported in subjects with ASD (Ochsner et al., 2005; Northoff et al., 2006). The above-mentioned brain regions are involved in many impaired mental functions seen in the ASD group, such as difficulty in decision making (Luke et al., 2012), lack of identification with self (Northoff et al., 2006), cognitive deficits (Baron-Cohen et al., 1985) and problems in self-referential thought (Ochsner et al., 2005) and mentalizing (Frith and Happe, 1993; Frith and Frith, 1999; Gallagher and Frith, 2003; Kana et al., 2009; Gotts et al., 2012; Schurz et al., 2014). Especially, the medial prefrontal cortex and posterior cingulate cortex are implicated in the theory of mind network, a key symptomatic feature of patients with ASD. Therefore, we assert that the under-connectivity in the medial prefrontal cortex and posterior cingulate cortex may cause the social functioning impairments in ASD.

### Inter- and intra-hemispheric connectivity

Using inter-hemispheric correlation, Anderson et al. showed a lower inter-hemispheric correlation in the sensorimotor cortex, superior parietal lobule, and frontal insula in subjects with ASD compared to that in controls (Anderson et al., 2011). Moreover, the mean corpus callosum volume in the ASD group was significantly smaller than that of TDCs. Although, both corpus callosum volume and gray matter inter-hemispheric functional connectivity were significantly reduced in autism, no direct relationship was observed between them, suggesting that the structural and functional imaging measure different aspects of inter-hemispheric connectivity (Anderson et al., 2011).

In our study, the inter-hemispheric differences between the two groups were computed by the difference in cFCDs between the two groups. Regions showing lower cFCD in the ASD group were the posterior cingulate/precuneus, somatosensory areas, parahippocampal gyrus, and angular gyrus. However, the mean cFCD in the ASD group was not significantly different from that of TDCs and the mean cFCD in the ASD group was even higher than that of TDCs after normalization. On the contrary, the examination of the mean iFCD showed that it is significantly reduced in the ASD group compared to that of TDCs. As intra-hemispheric under-connectivity contributed more to the overall results of our study, global under-connectivity in patients with autism may be a result of intra-hemispheric under-connectivity rather than inter-hemispheric under-connectivity. This result is consistent with that of a prior study showing altered intra-hemispheric connectivity in the autistic brains (Minshew and Williams, 2007). In reality, intra-hemispheric connectivity has been less studied than inter-hemispheric connectivity, due to the complex and time-consuming processes needed to calculate these values. However, our results suggest that the models of under-connectivity in autism should consider intra-hemispheric as well as inter-hemispheric connectivity for more sophisticated understanding of how connectivity abnormalities would be determined by interactions between the intra- and inter-hemispheric functional connectivity in the ASD group.

### Correlation between connectivity and clinical measures

We found that there were significant correlations between the average of gFCD, cFCD, and iFCD values in the lingual/parahippocampal gyrus and ADOS-RRB scores (Figure 5). The lingual/parahippocampal gyrus is connected with the amygdala and limbic structure in the brain, and is believed to play an important role in the processing of visual information about parts of the human faces (McCarthy et al., 1999) and processing high-emotion words or images, identifying visual scene and social context, and paralinguistic communication (Epstein and Kanwisher, 1998). These functions are the main areas in which individuals with ASD suffers from severe impairment, and the abnormality in the lingual/parahippocampal gyrus has been reported in individuals with autism (Weng et al., 2010). The significant correlation between gFCD and iFCD in the lingual/parahippocampal and ADOS-RRB scores can be explained by a theoretical framework provided by the salience landscape theory (Ramachandran and Oberman, 2006). Although autism is mainly considered a social disability, it also has non-social features, such as restricted and repetitive behavior/interests and sensory hypersensitivity. The salience landscape theory provides a compelling explanation for this symptom dimension of autism, suggesting that the altered connections between the limbic system and the sensory areas could cause extreme emotional responses and autonomic hyperactivity to the surrounding environment in patients with ASD, and repetitive behavior has a compensatory calming effect by reducing the child's autonomic arousal. The lingual/parahippocampal area is adjacent to, or a part of the limbic system. Therefore, our result suggests that the functional connectivity of this area might be altered in a different way in individuals with ASD, and these alterations may explain the restricted and repetitive behaviors in the ASD group.

### Differences in a recent study using ABIDE dataset

Recently, a study using a large dataset from ABIDE was published with 360 male subjects with ASD and 403 male TDCs (Di Martino et al., 2014). They used four different imaging analysis methods. Among the four methods, the degree centrality was similar to gFCD used in this study. However, their results using the degree centrality were different from our results, in that the ASD group showed over-connectivity in the right middle frontal gyrus. In the right posterior cingulate cortex, no difference was observed between the two groups according to the degree centrality analysis, but in our analysis, the ASD group showed under-connectivity in the posterior cingulate cortex. Even though the degree centrality method and FCD methods are both data-driven voxel-wise methods, several factors may explain such different results. The degree centrality method used eight times lower resolutions in voxel dimensions (4 × 4 × 4 mm) (Zuo et al., 2012) than our method (2 × 2 × 2 mm). Their sample size was smaller than our sample size; they used only male subjects, whereas we used all subjects, including females and adults.

## Limitations

There are several potential limitations in this study. First, the age of the subjects with ASD and TDCs are widely distributed from childhood through adolescence to adulthood. We considered only the average FCDs of all subjects from these broad age groups. Although we found average patterns of functional connectivity in the ASD group compared to those in the TDC group after setting age as a covariate, we may have missed age-related changes in the functional connectivity of the ASD group. Brain structure changes during human development (Sowell et al., 1999, 2003). In particular, adolescence is a time of human brain maturation as remarkable physical and behavioral changes occur (Paus et al., 2008; Koyama et al., 2011). Thus, future research should focus on the age effects on the neural system in ASD. The second limitation is that the FCD mapping analytical method found cortical hubs, but we did not examine which regions were strongly connected with those hubs after group comparison. This approach may require higher computational power. Third, the inconsistent findings regarding over- vs. under-connectivity in the brains of patients with ASD might be due to individualized alterations in the functional connectivity patterns, and the group comparison study like ours may not have taken functional idiosyncrasy into consideration as a possible source of these discrepant findings in functional connectivity (Hahamy et al., 2015).

## Conclusions

In conclusion, we observed abnormalities of global functional connectivity in ASD by applying FCD mapping on data from 517 TDC subjects and 458 patients with ASD from the ABIDE datasets. We found regional under-connectivity in ASD by comparing of intra-hemispheric, inter-hemispheric, and global functional connectivity. Our findings suggest that a deficit of non-social functioning process in ASD, such as restricted and repetitive behaviors and sensory hypersensitivity, might be determined by both the inter- and intra-hemispheric functional disconnections in the posterior limbic and sensorimotor regions.

## Author contributions

Equally contributed first authors: JL, SK. Conceived and designed the experiments: JL, SK, KC. Performed the experiments: JL, SK, KC. Analyzed the data: JL, SK. Contributed reagents/materials/analysis tools: JL, SK, EK, KC. Wrote the manuscript: JL, SK, EK, KC.

### Conflict of interest statement

The authors declare that the research was conducted in the absence of any commercial or financial relationships that could be construed as a potential conflict of interest.
